# Rethinking the Body in the Brain after Spinal Cord Injury

**DOI:** 10.3390/jcm11020388

**Published:** 2022-01-13

**Authors:** Erik Leemhuis, Valentina Giuffrida, Maria Luisa De Martino, Giuseppe Forte, Anna Pecchinenda, Luigi De Gennaro, Anna Maria Giannini, Mariella Pazzaglia

**Affiliations:** 1 Department of Psychology, Sapienza University of Rome, Via dei Marsi 78, 00185 Rome, Italy; erik.leemhuis@uniroma1.it (E.L.); valentina.giuffrida@uniroma1.it (V.G.); marialuisademartino@gmail.com (M.L.D.M.); anna.pecchinenda@uniroma1.it (A.P.); luigi.degennaro@uniroma1.it (L.D.G.); annamaria.giannini@uniroma1.it (A.M.G.); 2 Action and Body Lab, IRCCS Fondazione Santa Lucia, Via Ardeatina 306, 00179 Rome, Italy

**Keywords:** spinal cord injury, neural plasticity, central nervous system, face representation, limb representation, phantom limb, neuropathic pain, rehabilitation, disembodied, deafferentation, body representation, somatotopy

## Abstract

Spinal cord injuries (SCI) are disruptive neurological events that severly affect the body leading to the interruption of sensorimotor and autonomic pathways. Recent research highlighted SCI-related alterations extend beyond than the expected network, involving most of the central nervous system and goes far beyond primary sensorimotor cortices. The present perspective offers an alternative, useful way to interpret conflicting findings by focusing on the deafferented and deefferented body as the central object of interest. After an introduction to the main processes involved in reorganization according to SCI, we will focus separately on the body regions of the head, upper limbs, and lower limbs in complete, incomplete, and deafferent SCI participants. On one hand, the imprinting of the body’s spatial organization is entrenched in the brain such that its representation likely lasts for the entire lifetime of patients, independent of the severity of the SCI. However, neural activity is extremely adaptable, even over short time scales, and is modulated by changing conditions or different compensative strategies. Therefore, a better understanding of both aspects is an invaluable clinical resource for rehabilitation and the successful use of modern robotic technologies.

## 1. Introduction

Spinal cord injury (SCI) is a devastating neurological injury that mostly affects young individuals, severely limiting their normal life activities. SCIs are commonly attributed to sudden trauma, acute/chronic disease processes, or degeneration of the vertebrae [1]. Unfortunately, there is no satisfactory treatment for SCI victims yet. The degree of impairment depends on the level and entity of the lesion, with partial or complete loss of sensation and loss of voluntary movements of the body below the level of the lesion. Early clinical treatment at the primary lesion site, such as surgical decompression, is frequently associated with significant clinical improvements [2]. However, the injury is also immediately accompanied by a sustained cascade of biological events [3,4,5], termed “secondary injuries.” These dynamic effects lead to neuroplastic changes below and above the lesion throughout the central nervous system (CNS), including the brain [4]. Therefore, understanding the subsequent reorganization entails placing emphasis on the specific damaged tissues, studying the ensuing events from a pathophysiological perspective [5]. Alternatively, the rehabilitation-based perspective, in its widest and most multidisciplinary sense, focuses on enabling the body to recover and regain its ability to interact physically and socially with the world [6]. Here we explore yet a third method, not considered as an alternative but encompassing different approaches, whereby the body and its representations in the nervous system, act as a bridge between neurobiological and clinical/behavioral outcomes.

We provide a comprehensive yet concise summary of the current knowledge on how SCI impacts the representation of the entire body, both above and below the injury. The first section summarizes the main structural and functional neuroplastic events that typically occur throughout the CNS following SCI and that unfolds at different spatial and temporal scales. The second section discusses the most relevant studies relating to body representation for the three main parts of the body: (i) lower limbs, (ii) trunk and upper limbs, and (iii) head and face. Only a better understanding of the neurobiological and clinical/behavioral outcomes will allow the development of appropriate therapeutic strategies. 

## 2. Neural Plasticity following SCI

**Figure 1:**
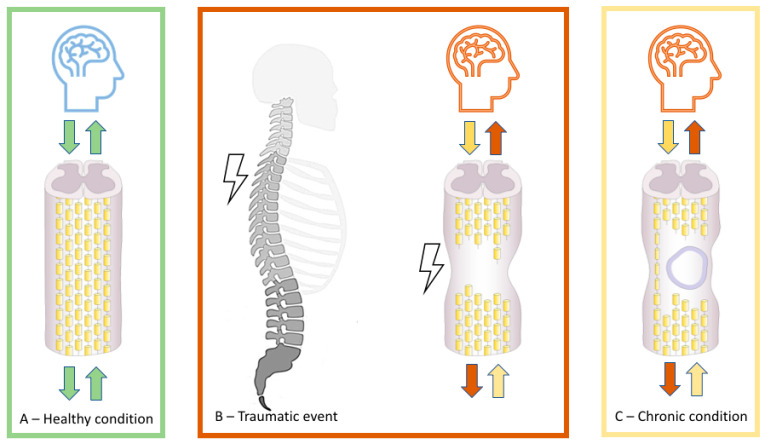
Schematic representation of the pathophysiology of a normal (**A**) and an injured spinal cord (**B**,**C**). The initial mechanical trauma to the spinal cord (**B**) initiates a secondary injury cascade that is characterized by edema, hemorrhage, inflammatory cells (**B**), and the persistence of scar tissue (**C**), which can lead to further cell death, demyelination, and neurological impairments in orthograde and retrograde directions, including brain areas.

At the neural systems level, an altered signal initiated at the lesion’s origin in the spinal cord spreads until the endpoint of the sensorimotor tract [7,8,9] in the cortex causing changes at all levels of CNS circuitry (i.e., the spinal cord, brainstem, and brain). These dynamic processes in the CNS circuitry involve modification of cortical somatotopy, requiring changes in body self-perception [10]. The immediate damage to neurons, axonal circuits, and to the tissue in the injured area cause the formation of a glial scar that limits tissue loss but also acts as a barrier for axonal growth [4]. Neurodegenerative process occurs along the dorsal columns and in the descending corticospinal tracts presumably, driving the activity of large cortical networks [11,12,13]. Neuroimaging techniques are the most powerful methods for mapping on human brain the consequences of “disconnected” spinal segments. These spinal changes (Figure 1) are the main trigger of neuroplasticity in neural centers supraspinal can lead to abnormal cortical excitability, altered connectivity and neural changes [13,14,15,16,17,18,19,20,21,22,23,24]. Specific brain morphometric differences with a reduction in white matter and gray matter, involve but are not limited to, the primary motor [11,13] and sensory cortices [11]. Resting-state functional magnetic resonance imaging studies have revealed that the strength of intra- and inter-hemispheric functional connectivity within sensorimotor cortex is dependent on ascending and descending altered signals from spinal and immediately supraspinal structures [10,13]. Brain activity alterations [23,24] can be recorded even in incomplete SCI injuries [25] and are correlated with the number of “disconnected” spinal segments [21,24,26,27,28]. Progressive atrophic, microstructural, and biochemical changes after SCI has also been associated with altered motor and sensory body behaviour [29,30,31,32,33]. Given the reduction in body efferent and afferent signals, the entire cortex is also exposed to reduced functional connectivity, that constantly includes different areas [34,35,36,37]. Structures, such as the fusiform gyrus or the orbitofrontal cortex, may also be at play in anatomical and functional reorganization also occurs in the sensorimotor cortex [38]. However, it is possible that despite the morphometric changes of sensorimotor activity after SCI, somatotopically typical representations of the paralysed and sensory deprived body parts can be initially preserved but deteriorates across time.

In addition, cortical reorganization after SCI has also been associated with neuropathic or phantom limb pain [37,38,39]. The onset and evolution of pain in SCI and its effects on the nervous system remain unclear. Huynh, et al. [39], reviewed imaging studies to assess supraspinal neuroplasticity differences between groups of SCI patients with and without neuropathic pain in the resting condition. They found changes in the concentration of metabolites in sensory and pain-related areas, such as the thalamus and the anterior cingulate cortex, that depended on the presence and intensity of neuropathic pain [40,41]. Furthermore, grey matter volume alterations in the same areas have also been observed in SCI patients with neuropathic pain [23]. Differences in gray matter volume of motor and somatosensory primary cortices indicate other possible sites related to neuropathic pain [23]. Finally, differences associated with neuropathic pain have been observed in activity patterns within the insula, making it a possible target for future clinical research [41].

Important information on the over time evolution of SCI also comes from the perceptual symptoms reported by patients. Phantom limb sensations are common after SCI [42], both for upper and lower limbs (depending on the lesion level), but less well-characterized and studied than in amputations and other neurological deafferentations such as brachial plexus avulsion [43,44,45,46]. Understanding such symptoms and their spontaneous and rehabilitation-driven evolution may help reveal the brain-body coupling mechanisms, improving clinical outcomes. In this context, different studies have clearly shown a seemingly contradictory contribution of processes promoting stability, remapping, and reorganization. To integrate the two perspectives, Muret and Makin (2020) have recently proposed that homeostasis can be scaled up from an intra-cellular level to a neuronal network level. That is, the representation of adjacent body parts in the somatosensory Penfield’s homunculus may overlap and borders fade. Finally, the loss of sensory input alters normal lateral cortical inhibitory processes, emphasizing latent activity in the deafferented areas as well as homeostasis [47].

This analysis introduces new factors that may help disentangle the complex dynamics of brain adaptation post-SCI and may assist in understanding phenomena such as phantom limbs or the long-term somatotopic stability of the deafferented body.

## 3. Body Reorganization and Body Stability of Somatosensory-Motor Topographies

Currently, the most effective technique for addressing body-brain disconnection in SCI is brain activity registration [48] during three main activities: mental imagery, sensory stimulation, and motor activity. However, the mechanisms through which the brain interprets and fails to record the signals from affected and non-affected body parts remain controversial. We examine the evidence in favor of somatotopic rearrangement as a possible representational rule underlying the loss of extension, reductions, or absence of activity in the affected body, while considering how movements and sensations of the body are expanded and shifted.

### 3.1. Reorganization and Stability of the Lower Limb Representation

The traditional view of this problem suggests that severe sensorimotor impairment should correspond to shifts, loss of extension, and reductions in cortical thickness and/or activation in lower-limb corresponding areas of the brain. However, this represents an over-simplification caused mainly by over-interpreting the results of early studies in the field due to the clinical need to categorize sensory and motor residual functions. According to the American Spinal Cord Injury Association (ASIA) classification, a complete SCI lesion is classified as AIS Grade A, meaning that the patient does not feel any sensation—or cannot produce any motor activity—in the part of the body below the lesion. Meanwhile, an AIS Grade B lesion means that while body sensations from below the SCI are spared, complete motor loss is present. AIS grades C–E describe different levels of sensorimotor impairment. However, more recent evidence suggests that at least one more level exists between AIS grade A and B; over 30 years ago, such a condition was known as a discomplete lesion whereby at least sensory information was able to reach the brain, even in the absence of a patient’s awareness [49]. Importantly, recent studies have shown that sensory connections are spared in approximately half of the lesions considered to be complete [50,51]. The purpose of these brief considerations is to highlight the risk of relevant biases that occur in most studies regarding the consequences of an SCI on the CNS, in line with a recent review [52].

A procedure seeking to identify changes in the cortical representation of the lower limbs consists of simple sensory stimulation. Gentle toe brushing of patients with clinically complete SCIs (AIS Grade A) was used to evoke S1 activation, making it possible to discriminate between complete and incomplete lesions [50]. In 11 of the 23 participants with complete lesions, significant S1 activation was produced by toe brushing, which was not correlated to the presence of neuropathic pain. Furthermore, the location did not differ between the subjects with discomplete lesions and healthy control participants. Moreover, activity was recorded in the contralateral S2, contralateral thalamus, and ipsilateral cerebellum, without any significant location differences. A subsequent study confirmed how different types of sub-lesional sensory stimulations (touch and nociception) correlate with brain activation in the expected contralateral somatosensory areas [51]. Activation was also recorded in the bilateral S2 region and contralateral Brodmann area 5, the insula, and anterior cingulate cortex for nociceptive stimuli.

Depending on the level and severity of SCI, researchers have used various experimental paradigms, including motor activity, motor attempt, and mental/motor imagery tasks, to study lower limb-related brain activity in humans. For example, Sharp et al. [53] using fMRI, offered important insights into the main characteristics of lower limb-related brain activity changes during ankle dorsiflexion. In patients with incomplete spinal cord lesions, the authors found a correlation between higher activation levels and more severe impairment of the lower limbs. Moreover, the chronicity of an SCI plays a major role in the exacerbation of these changes, especially in the supplementary motor area and left frontoparietal regions, pointing to the occurrence of a process of adaptation. In this study, the researchers observed enhanced recruitment of the expected correct sensorimotor areas (i.e., non-dominant) during voluntary movement of the right ankle, and a higher level of brain activity correlated with a higher impairment level. Recently, new studies have been conducted on body-related cortical reorganization that have explored the impact of mental imagery repetitive tapping task of both upper- and lower-limbs in patients with complete SCI [54]. When compared to healthy controls, the blood oxygenation level-dependent (BOLD) response in SCI patients showed both stronger and weaker responses to upper-limb mental imagery tasks. Healthy controls showed strong signals in the left middle frontal gyrus, left anterior cingulate, and left medial frontal gyrus. Patients with complete SCI have stronger activation in the left inferior parietal gyrus, left precentral gyrus, and left postcentral gyrus. A stronger BOLD response in the SCI group was also evident in the right globus pallidus, right thalamus, right superior temporal gyrus, right inferior parietal gyrus, and right precentral gyrus. In the lower-limb version of the imagery task, controls produced stronger signals in the right anterior cingulate, right medial frontal gyrus, right superior temporal gyrus, and right superior frontal gyrus. However, patients with SCI demonstrated more intense activity in the left lingual gyrus and right globus pallidus [54]. The authors found a negative correlation between time since injury and signal intensity in the left precentral gyrus and left lingual gyrus, suggesting that this may be a consequence of compensatory mechanisms triggered by motor and sensory loss. Therefore, a generalized compensatory process may be found in the stronger activation of visual and auditory subcortical processing areas for both upper-and lower-limb motor imagery tasks. Despite the contradictory findings in activation intensity and patterns in fMRI studies, it appears appropriate to consider the cortical structural representation of the areas of the body below the lesion as mostly stable throughout both the acute and chronic phases. When assessed, somatotopy of the lower limbs––specifically the ankle and toes––corresponds to that of healthy controls. However, in terms of activation patterns and intensity, while it remains too early to provide precise answers, compensatory processes likely play a fundamental role in the recruitment of contralateral sensorimotor areas as well as in the involvement of more distant cortical and subcortical structures.

### 3.2. Reorganization and Stability of Upper Limb Representation

The upper body, specifically the upper limbs, offers a wider range of experimental opportunities to assess changes in body representations in the brain following a SCI, when compared to the lower limbs. TMS is a non-invasive brain stimulation technique that produces different effects depending on the target area. For example, stimulation of the primary motor cortex with TMS produces motor-evoked potentials that can be recorded in the muscles [55]. TMS has long since been used to explore the effects of deafferentation and has helped confirm changes in the cortical representation of the body showing how, in SCI patients, to produce motor evoked potentials of muscles surface and position of the stimulation site can be different than in healthy controls [56]. As previously mentioned, the results from past studies are not entirely consistent; however, differences continue to be found in the extent, intensity, and pattern of brain activity related to movements of the upper body in the case of SCI. For example, in their fMRI research, Curt et al. found a substantial stability of the somatotopic organization of the upper limbs in M1 during a controlled movement task of the upper limb and tongue free movement. However, the pattern and extent of activation differed from those of healthy participants [36].

In the primary motor area, only the movement of the fingers produced a change, which consisted of more extensive activations that expand medially and laterally compared to that in the controls. Movements of both the wrist and elbow in SCI patients are related to greater activation of non-primary cortical areas involved in movement, such as the supplementary motor area, the dorsal premotor area, the primary somatosensory cortex, and the cerebellum. Just a few years earlier, a PET study, using a hand movement task to control a joystick, identified, albeit in less detail, differences in activation patterns, also differentiating paraplegic and tetraplegic participants in comparison with healthy controls [21]. The activation areas shared with healthy subjects, in the case of SCI, presented greater and more extensive activations for both paraplegics and tetraplegics, which also involved cortical areas normally associated with the legs. Furthermore, both SCI groups also showed activation of the parietal, cerebellar, and thalamic areas. Counter-intuitively, the paraplegics showed more intense and extensive activity, which was explained by the greater sensorimotor impairment of the hand in tetraplegics. The shift in hand-related somatosensory areas was subsequently confirmed in larger groups of patients with SCI using fMRI during a tactile stimulation of the mouth, fingers and toe [24]. This shift mainly occurs towards the medial areas that correspond to the legs, or, more generally, the sub-lesion part of the body. This phenomenon is particularly affected by the area corresponding to the little finger, which extends its activation area by 13 mm towards the cortical areas directly involved in SCI and therefore is “disconnected” from the body. It is noteworthy that the loss of gray matter that occurs in most of the deafferented S1 areas was not seen in individuals in whom hand-related activity was extended. This is likely due not only to the exceptional recruitment of dormant neuronal populations found in the short term after the injury [57] but also to a real trophic process of sprouting that leads to an increase in lateral cortical connections [24]. Using TMS, Fassett, Turco, El-Sayes and Nelson [56] found alterations in somatotopic organization in the bilateral primary motor cortex of the biceps, flexor carpi radialis, and abductor pollicis brevis in participants with an incomplete cervical SCI. While the overall somatotopic organization between the parts studied does not present substantial differences between SCI patients and healthy controls, the entire region is medially shifted in SCI. A further change concerns the extension of the cortical territory relating to the abductor pollicis brevis, which is more accentuated than that in healthy participants. Researchers attribute these aspects to two major possible factors: learning of motor skills related to the need to adapt to new physical conditions and compensatory mechanisms of plasticity due to the need to optimize the neural output necessary to control muscles whose functionality is compromised [56]. In some tetraplegic patients, finger movement is no longer possible, depending on the level of the lesion, while the arms can still be controlled. However, surgical transfer of the distal brachioradialis tendon to the flexor pollicis longus can restore the pinch grip of the flexion of the thumb [58]. After a long training period, patients learn to move their thumb, controlling a muscle normally used only in elbow movements. Interestingly, the movement change corresponds to a change in the areas activated in motor cortices [59]. Furthermore, researchers have shown that in patients with SCI, cortical activity related to the brachioradialis muscle shifts from the elbow area (before the surgical tendon transfer) to the wrist-related area. This suggests that the motor cortical areas and their connections remain intact, even after prolonged disconnection from the corresponding body areas.

In addition, the ability of plastic reorganization is maintained to allow these areas to participate in long-lost motor activity (i.e., at the time of spinal injury), even when using a muscle that was previously not directly involved in this movement. After some time, in the same group of patients, it was possible to study the morphological changes in the cortical areas involved in thumb movement. That is, the primary motor cortex showed a general condition of atrophy, but when specifically considering only the area “reactivated”, because of the tendon transfer procedure, there was no difference in cortical thickness between tetraplegic patient and healthy controls [60].

### 3.3. Reorganization and Stability of Face Representation

Dorsal column lesions [61,62,63,64] or dorsal root transections [65] of the spinal cord in non-human primates are prominent models for large-scale face cortical reorganization of the primary somatosensory cortex. During this reorganization, the facial inputs for hand deafferented representation expand by 7–20 mm [61,63,64,66], shifting the face/hand map boundaries [63].

In fact, somatotopic remapping processes occur with the establishment of new connections between the face and the deafferented hand region in the primary somatosensory cortex [67,68]. Reorganization is also seen in the ventroposterior nucleus of the thalamus [63,69,70,71] and in the spinal cord and dorsal column nuclei [72], despite the fact that selective somatotopic interference, cortical dynamics, and neuroplasticity, based on competitive interaction from a normally innervated territory, are common and widely accepted. However, there remains no objective evidence supporting a link between neural topographic substitution and peripheral loss in humans, as has been observed in monkeys. Brain-imaging studies of humans show contradictory findings, reporting possible reorganization of the cortex after SCI with expansion of the representation of the face into the hand region [38,47,73]. Specifically, it has been shown that, in patients with cervical lesions, tongue movement is displaced medially, posteriorly, and superiorly (12 mm) toward the adjacent cortical deafferented hand area compared to that in controls, with a strong correlation between displacement and the level of SCI [73]. Similar cortical remapping was found by Corbetta et al. [37], who used visuomotor monitoring to assess a patient with cervical SCI. The patient had to synchronize his tongue movements with a video image, after which he received vibrotactile stimulation in the palm of his hand. They found stronger and more widespread BOLD responses in the patient than in the control subject. The activity of brain responses to tongue movement spread dorsally in the hand area and broadly in the second somatosensory cortex and frontal operculum. In addition, the SI/M1 face area was activated in the ventral precentral gyrus, central sulcus, and postcentral gyrus.

Overlapping fMRI scans of the SCI patient and the control revealed that the active facial region was similarly active in the two participants; however, in the SCI subject, it invaded the hand area. While reorganization patterns following SCI in humans suggest a topographical remapping in the region closer to the site of deafferentation, the tactile threshold required to elicit a neural response of the new face map, and whether reorganization of face representation occurs in higher somatosensory areas, remains unknown in humans after cervical SCI. However, behaviorally, greater susceptibility to the modified rubber hand illusion paradigm (applying sensory stimulation to the face with the observed touches on the fake hand) seems to drive tetraplegics’ sense of hand ownership [74], supporting topographic remapping of the face on the hand territory. Moreover, “somatotopic interference” of the face–hand is suggested by vivid and clear “phantom” limb experiences in SCI, which implicitly confirm topographical remapping of cortical territories following peripheral disconnection [75]. However, these previous studies were unable to determine whether such activity represents functional reorganization, supporting the sensorimotor hand. Therefore, some descriptions of face-hand remapping may be misleading [76]. For example, Curt et al. (2002) did not report the topographical reorganization of M1 but did observe increased activation during tongue movements [36]. In addition, fMRI studies found no apparent expansion in the somatotopic cortical representation during attempted face movement in individuals with SCI and revealed no significant differences when compared with healthy controls [36,77]. It is also noteworthy that advanced studies using MRI with VMB and DTI analysis [24], despite reports of cortical reorganization of the hand, found no evidence of a shift following tongue movements. It is worth noting that discrepancies between human and animal neuroplasticity arise from biological differences between species, including variations in the way different species experience their bodies when interacting with the world. Beyond these differences, many changes in the relevant functional networks may be related to the extent of neurological injury and the severity of the lesion, which determine the inconsistency in results for the heterogeneous sample. Time is another critical factor related to remapping, and longitudinal studies are required to understand the progressive development of processes that, by their very definition, extend across different time scales. Shift occurs at a minimum of one year following SCI, depending on the severity of the SCI [78] indicating less chaotic reorganization subsequent to the absence of hand inputs. Changes in cortical activity may lead to the convergence of metabolic and neural processes, such as aberrant signaling [79], altered cellular metabolism [80], axonal sprouting, and fluctuations in synaptic strength due to upstream reorganization [33]. Numerous subcortical changes also play a role in reorganization, specifically thalamic reorganization, in which facial afferents in the trigeminal nucleus may grow within the cuneate nucleus [62]. For example, a study by Kambi, et al. [81] sought to identify a causal relationship between all these changes and cortical reorganization in non-human primates. Their results showed that cortical reorganization following sprouting of chin input in deafferent areas does not affect reorganization. In contrast, when the cuneate nucleus was blocked by lidocaine, there were no longer any responses from the chin region, which had previously expanded into the hand region following stimulation of the chin. Therefore, it could be assumed that humans possess the same control of the cuneate nucleus. If so, brainstem could represent a starting point for controlling cortical reorganization of somatosensory cortex [81].

## 4. Conclusions

The studies discussed in this paper show that peripheral and CNS lesions can induce neural reorganization within the central somatosensory and motor/body representations. However, how the brain processes the flow of signals following a body-brain disconnection due to a complete SCI remains largely unknown. How deep severe neurological traumas affect typical sensory and motor signals of single body parts and their appropriate integration by brain mechanisms and structures is unclear. In fact, there are discrepancies in the observed brain activity changes in patients with SCI. This can be attributed to heterogeneity in the extent of the neurological injury and lesion severity, to disease duration, age, as well as to the degree of altered sensory and pain experience, to compensatory learning, and to exposure to rehabilitation [82]. Importantly, the mechanisms underlying SCI are multifactorial, and changes to a patient’s temporal dynamics and experiences as well as spinal and supraspinal modifications may be the main drivers of neuroplasticity in the sensorimotor centers [11,12]. Furthermore, reorganization of the neural system appears to represent a response to body pain [83,84,85]. The approach proposed in this paper offers new insight for the body rehabilitation and the use of assistive devices [84,86,87,88]. It proposes that it is advantageous to consider somatotopically head, upper-limb, and lower limbs body areas for focal stimulation, to boost the sense of embodiment and agency in body parts with reduced access to sensori-motor information [89]. Yet, this may produce positive residual responses, improving the effects of treatment rehabilitative [90,91].

## Data Availability

Not applicable.
